# Modeling Biology Spanning Different Scales: An Open Challenge

**DOI:** 10.1155/2014/902545

**Published:** 2014-07-17

**Authors:** Filippo Castiglione, Francesco Pappalardo, Carlo Bianca, Giulia Russo, Santo Motta

**Affiliations:** ^1^Institute for Applied Mathematics, National Research Council of Italy, Rome, Italy; ^2^Department of Pharmaceutical Sciences, University of Catania, Catania, Italy; ^3^Theoretical Physics of Condensed Matter, Sorbonne Universities, UPMC Univ Paris 6, 75252 Paris Cedex 05, France; ^4^UMR 7600 LPTMC, CNRS, 75252 Paris Cedex 05, France; ^5^Department of Mathematics and Computer Science, University of Catania, 95125 Catania, Italy

## Abstract

It is coming nowadays more clear that in order to obtain a unified description of the different mechanisms governing the behavior and causality relations among the various parts of a living system, the development of comprehensive computational and mathematical models at different space and time scales is required. This is one of the most formidable challenges of modern biology characterized by the availability of huge amount of high throughput measurements. In this paper we draw attention to the importance of multiscale modeling in the framework of studies of biological systems in general and of the immune system in particular.

## 1. Introduction

The language of mathematics has been extensively used to describe natural phenomena of the physical sciences in terms of models based on equations. The mathematical language allows logical reasoning over a representation of the physical entities involved in the phenomenon and makes possible to account for the observations made through experimentation.

In designing the mathematical model of a natural phenomenon the first and fundamental step is to define the mathematical variables that play a role in the phenomenon under investigations, according to the goals which the model is built for. For example, to calculate the decay rate of a certain protein, a variable to describe the changes of the protein concentration in the blood can be used. In this case the dynamics of the atoms and the ions is neglected and the information about the folding of the protein itself is lost. The origin of this oversight is related to the basic principle sometimes referred to as the* lex parsimoniae* most commonly known as the* Ockam's Razor*. “Pluralitas non est ponenda sine necessitate” in very simple words states that in the description of a phenomenon, the most useful model is the most parsimonious one in terms of elements used. In this regard, following up the above example, it makes little sense to describe the laws governing the forces accounting for the folding of the protein if we are interested in the half-life of the protein and we can estimate its decay rate by fitting a curve to a set of experimental data about the concentration in the blood of that protein.

William of Ockham was a Franciscan monk and logician who lived in the 14th century in a village of the English county of Surrey. At that time the principle of parsimony in describing and modeling a natural phenomenon was well reasoned. However, today the situation is a bit different. The* lex parsimoniae* is still valid and indeed is used when describing a phenomenon, but besides classical mathematical models allowing for an exact analytical approach, another modus operandi is now commonly employed [1–3]. This is what we can call the* synthetic approach* consisting in constructing a* replica or toy* of the studied system in terms of the most important identified elements and the laws governing the relationship among them. Actually this approach is not new at all. The “engineer” Leonardo Da Vinci used this approach to construct toy models of flight machines before attempting anything real-scale.

What is new today is that we can use digital computers to construct toy models of complex systems. Indeed extremely powerful CPUs can be instructed to execute algorithms representing entities and laws and all kinds of conceptual experiments on those entities and laws can be made. This “digital synthetic” approach is commonly referred to as simulation.

Today, when studying a certain natural phenomena, scientists first identify elements and basic laws governing the dynamics of the system then they represent them as data structures and algorithms and finally execute the algorithms to observe how the system evolves. The Ockam's principle is still valid and used in the first phase of this process but beyond that the parsimony is forsaken, and the complexity of the initial toy model is augmented by simply adding new entities and laws. Indeed, with little difficulty we can detail processes incorporating hypothetical or experimentally derived knowledge. We can even* compose* preconstructed models of different parts of the real system or arrange models describing reality at different scales of observation, thus constructing a multiscale model. Ockam's Razor has been extensively used in classical mechanics models generating a cascade of models of increasing complexity. An interesting example arises from models of fluid dynamics which consider first uncompressible nonviscous fluids in a linear regime to move toward more complex situations like boundary layers and turbulent regimes. Models including different regimes are still difficult to perform.

This holistic approach is what in modern biology is called* systems biology* [4]. In this regard, there is another important aspect that should not be left out from the whole picture: the contemporary data explosion deriving from genomic, transcriptomics, proteomics, and metabolomics studies consisting in high dimensional datasets produced by latest high throughput measurements methods [5]. Other types of data coming from modern microscopy and biological imaging contribute as well to the detailed description of the constitutive parts and basic structures of living organisms [6]. On that account, the challenge has its main feature in relating these datasets to higher-level phenotypic characteristics and computational multiscale modeling approaches are set to reveal quantitative mechanistic relationships between these various measurements [7]. For example, high throughput gene expression data can be used to infer knowledge of the intracellular activities that can be later ascribed to the behavior of cells in a higher-level description; for example, the expression of the gene GATA3 in CD4 T lymphocytes in certain experimental conditions gives indication about the differentiation state of these cells and ultimately on the Th1 or Th2 bias of the immune response [8]. This information is relevant to the construction of a mathematical model of the immune response.

Recently, the topic of multiscale modeling has been drawn a great deal of attention and is discussed in many articles and reviews [6, 9–15]. Similarly, the present paper aims at giving a meaning to the concept of multiscale modeling in the framework of studies of biological systems in general but with particular interest in the immune system. It provides a general introduction to the methodological issues of multiscale modeling avoiding pointing to a specific and well-defined method to deal with this matter. Indeed, while there are methods borrowed from other field (e.g., computational chemistry) that can be used in special cases, a well-developed mathematical framework that is general enough to account for the extremely large variety of biological phenomena, is still missing. Nevertheless, an interesting attempt in this respect is given in [16] together with two examples showing how to bridge different single-scale models. Extensive readings, including specific examples, can be found in the above-cited reviews and also in [17–22].

It is worth stressing that the important role that the environment has in the dynamics of complex physics and living systems is not considered in this paper. Therefore the contents of the present refer to closed systems.

## 2. From Micro to Macro: Scales in Biological Organization

**Figure 1:**
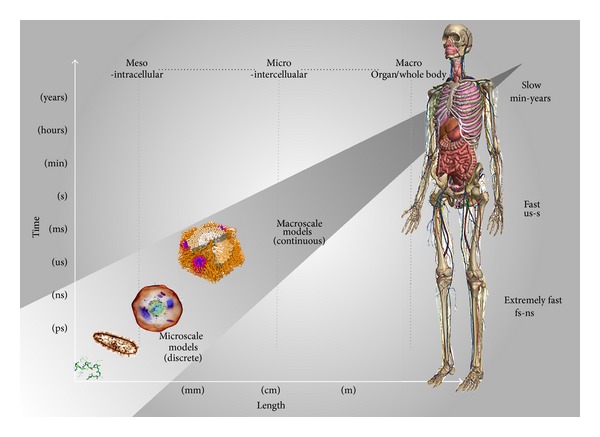
Multiscale models of the human body targeting complex processes span many time and length scales of biological organization. They cover a combination of discrete and continuous mathematical descriptions of different systemic components.

When “measuring” nature we choose a temporal and a spatial scale that is convenient to make a valid observation. The choice of the observation scale is an important step in science. In physics there is a somehow well-defined dividing line among different research areas based on the characteristic lengths of the systems studied and on the characteristic time of the phenomena under investigation. For instance, microphysics (e.g., molecular physics, atomic physics, nuclear physics, and particle physics) refers to areas of physics that study phenomena that take place at the microscopic scale (lengths < 1 mm). Similarly, in biology we can distinguish from molecular biology, microbiology, and cell biology looking at length scales below tenths of micrometers. Major levels of biological organization are regulated at scales of many orders of magnitude in space and time (see Figure 1), with space spanning from the molecular scale (10^−10^ m) to the living organism scale (1 m) and time from nanoseconds (10^−9^ s) to years (10^8^ s). In biology, while we can intuitively assert if a determined process involves cells, molecules, or organs, it is not so simple to identify values for the lengths at which we switch from one level to the next [6].

### 2.1. Single-Level Models

Roughly speaking, multiscale model is a composition of two or more “single” scale models representing the same phenomenon (or its parts) at different levels of descriptions. Even if the models we want to combine share the level of description, the manner in which the components are put together, namely, how the variables should be linked together, is a challenging part. For example, a simple model that describes the HIV infection of T helper lymphocytes may also take into account the coinfection of antigen presenting cells like macrophages and dendritic cells. Adding this new cell compartments to the original simplistic model introduces the problem of describing the immunological mechanisms of activation of the adaptive immunity by the innate one; in particular, the macrophages and the dendritic cells are both virus target and main actors of T helper priming.

Moreover in biological phenomena complexity arises not only from the action of many independent actors, like in social science, but also from the fact that changes at lower scales modify the way in which those actors will play at higher scale. For instance DNA modification in a cell may change the cell in a tumor cell which then duplicates much faster than a normal one changing the overall scenario both at cellular and at tissue level. In most biological models this “vertical” or “interscale” complexity must be taken into account.

In the study of complex phenomena involving the immune system in pathological conditions, a unified view is necessary to reach a comprehension of the various mechanisms in action and of the causal relationships among different immune system components as well as repercussions on different anatomical parts [7]. More than for other complex systems, the distributed nature of immune system functions evidences the need of an integrated approach. The evolution of a disease like diabetes or cancer [23] is representative of this fact.

As already mentioned, mathematical models that try to describe such mechanisms, usually fix the spatial and temporal scale and describe the system with a mathematical or computational (i.e., algorithmic) formalism [11, 12, 14]. Computers do the rest as they provide the dynamics by executing (resolving) the rules just described in the mathematical formalism. The whole dynamics depends on parameters and initial conditions so that one generally attempts hypothetical scenarios by modifying those initial conditions to get a feeling of the systems behavior [20, 22]. This process leads itself in discovering new knowledge. However, the problem is that the real system is in general not isolated hence a local description is not sufficient to disclose crucial mechanisms. It comes quite clear that one of the reasons why biological phenomena are intrinsically complex is because they are influenced by variables that are outside a single level of space/temporal description. Moreover the collective behavior cannot be simply inferred from the behavior of its elements and the alteration of only one element or one interaction reverberates on the whole system. Finally a global organization emerges from the interacting elements (emergent behavior), which does not exist at the individual elements level.

## 3. Top-Down, Bottom-Up, or Middle-Out?

It should be noted that experiments are done at many scales, ranging from single molecules or proteins to whole organs and organisms, and therefore, experimental information exists at different scales. Therefore, relying on different experimental data, a model can be formulated using two main approaches, that is, top-down or bottom-up [24, 25]. If one chooses to take into account the individual elements and their interactions, studying the resulting biological system as a consequence of the emergent behavior of its single components, then the bottom-up approach takes place. For example one can model the different immune system entities composed of cells and molecules to simulate the immune response against a specific pathogen, or one can use the cells as the basic elements and study tissue-level properties as results of the interactions of the cells. The advantage of this type of approach is that it is adaptive and robust, in the sense that if the available biological knowledge varies, one can adapt the new knowledge to the specific components of the model, in a very selective way. Moreover this kind of approach is suitable for studying the emergent properties of systems consisting of a large number of interacting elements. The intensive computer power required is the main disadvantage for the bottom-up approach and can be sometimes even prohibitive. Besides, the model itself can become too complicated to be controlled.

Instead, one can decide not to look straight into the details of the individual elements, but to consider the system at the macroscopic level, using experimental observations as guidelines during the formulation of the model. This is the case of the top-down modeling approach. For example, to keep on with the same example above, one can decide to model the immune system response against a specific pathogen ignoring the specific type of cells and their properties and modeling the global effect of population of cells, based on whole-cell experimental recordings. The clear advantage of this approach is that it is relatively simple. On the other hand, the flexibility and the robustness of the model are less evident compared with the bottom-up approach. Moreover, it should be highlighted that the variables and parameters in these models are largely phenomenological without direct connection with detailed physiological parameters. Due to this reason, it may sometimes happen that the top-down approach does not correctly reveal the actual responsible mechanism, for example, when there are multiple mechanisms for the same behavior or a single mechanism resulting in multiple effects. When existing components have to be integrated with some new part a third design principle, named “middle-out,” is used [26]. This paradigm promotes the integration of organs' models at different scales without posing limitations to the level of details each single component should be equipped with (recall the* lex parsimoniae*). In this regard, it must be emphasized that multiscaling is not about sophistication but rather poses a different challenge, that of the model integration.

## 4. Multiscale Modeling of Biological Properties and Functions

Spanning from the lowest scale to higher levels, different modeling techniques can be chosen [27]. For intracellular scale, the modeling technique tries to give a detailed description of the molecular processes happening inside the cells. Using experimental data, these kinds of models make use of the differential equation description to forecast the molecular dynamics of specific cellular pathways. Changes in the molecular concentrations are described by these models by mass action or Michaelis-Menten kinetic rate-law equations.

The Belousov-Zhabotinsky reaction represents a good example of a bidomain model that depicts a phenomenon beginning from the microscopic dynamics at a lower space scale, that is, wave propagation in reactive media. In its simple form it may be comprehended in terms of the following representation [28] including an autocatalytic reaction *A* + *Y* → *X* + *P*, *X* + *Y* → 2*P*, *A* + *X* → 2*X* + 2*Z*, 2*X* → *A* + *P*, and *B* + *Z* → *hY* + *Q*, where the variables represent concentrations of specific molecules (e.g., bromomalonic acid or carbon dioxide) and *h* is a constant. Translated to ordinary differential equation the system is *dX*/*dt* = *AY* − *XY* + *AX* − 2*X* ^2^, *dY*/*dt* = − *AY* − *XY* + *hBZ*, and *dZ*/*dt* = 2*AX* − *BZ*, where *A*, *B*, and *P* are held constant. The multiscale property of this model is found in the occurrence of the wave at a level that is above the one chosen to describe the phenomena, that is, the molecular level of the reactants.

The main difficulty is represented by parameter identification: the experimental estimation is often made in isolated systems that, by definition, do not permit generalization to the real case. If the interacting entities in a system to be modeled can be thought as homogeneous, then the most common choice is the use of ordinary differential equations. If the space is variable, then partial differential equations can represent a better technique [29].

In the case of intracellular models that consider small number of entities, microsimulation can represent an alternative to differential equations. The authors in [30, 31] proposed the Gillespie algorithm many decades ago. It allows simulating with a good accuracy chemical or biochemical systems of reactions generating statistically correct trajectories as possible solutions of a stochastic equation.

At a higher level of description, tissues or whole organs are modeled in two different ways: either as functional compartments or system units or as a collection of microscopic components (e.g., cells). In the first case rather than specifically model the organ, one can simply use the known input-output relationship as a black box. This relation is typically derived from experimental data or published results and ultimately developed by differential equations. These kind of phenomenological models aim at reproducing the observed behavior instead of trying to give an explanation. The modeling paradigm based on a collection of microscopic components intends to typify a tissue as an array of individual units (i.e., cells) exchanging signals with the environment. Examples of these multicellular systems have been originally developed to study the growth of solid tumors [32, 33] and have later on been applied to simulate the function (the regeneration) of complex organs like the liver [34].

An interesting example of a well-devised multiscale model has been developed in the framework of the hemodynamics [35, 36]. The problem deals with a detailed description of the fluid-dynamics of the blood, by mean of numerical integration of the Navier-Stokes equations, to cope with postoperative hemodynamics issues in congenital heart diseases, artery shunts, or similar heart surgery. In hemodynamics, local phenomena, such as the perturbation of flow pattern in a specific vascular region, are strictly related to the global features of the whole circulation. However, dealing with whole circulation using Navier-Stokes equations would be not just useless but rather impossible. The method proposes an interesting multiscale geometrical model where a local, accurate, three-dimensional description of blood flow by means of the Navier-Stokes equations in a specific artery (the region of surgical interest) is coupled with a systemic, zero-dimensional, lumped model of the rest of the circulation system [37]. What makes the example peculiar is the use of lumped models as those extensively used in electrical engineering that resort to simplified models in place of complex description of other system parts.

Another methodology worth to be mentioned is the one using “state transition diagram” [38, 39] which aims to solve the problem of heterogeneity and multiscale modeling and the link between mathematical and computer models [40]. This methodology, massively used in theoretical computer science and software engineering, describes the behavior of heterogeneous entities by means of (deterministic or probabilistic) finite state automata. Since each state of the automaton represents a “situation” related to a level of description, one could in principle set out a multiscale model as a combination of automata. However, since the number of states resulting from the combination of even simple models tends to be very large, this methodology does not seem to be of practical use.

If the interest is on simulating a whole cell, then several projects can provide useful hints (e.g., virtual cell [41], e-cell [42, 43]), whereas efforts aiming at simulating whole physiological systems or organs are, for example, models of the heart [18], of the liver [44], and of the skeletal system [45]. Other efforts aim at creating computational platforms suite to integrating various physiological processes by integration of different mathematical and computational models [46]. The approach is based on the principle that, in biology, there is no privileged level for the description of a certain phenomenon and that the interlevel causal relationships are driven by interactions between multiple levels [47–50].

It is worth stressing that the modelling of complex biological systems requires a completely different treatment with respect to the inert matter. Indeed the entities constituting the biological systems, which usually operate out-of-equilibrium, interact among themselves and with their outer environment and are able to perform individual strategies that modify the microscopic interactions among the entities composing the system [51].

Recently the kinetic theory has proposed an alternative approach for deriving macroscopic equations from the dynamics delivered at the mesoscopic scale: the asymptotic method. Accordingly, this method consists in deriving macroscopic equations by suitable limits of Boltzmann-type equations related to the statistical microscopic description; see the book [52], the paper [53], and the references cited therein. The first step in the development of asymptotic methods is the choice of the time-space scaling. Different types of scaling lead to different types of equations. After the assessment of the scaling, the distribution function is expanded in terms of a small dimensionless parameter. Finally the asymptotic limit is performed under suitable technical assumptions. Specifically parabolic (or low-field) scaling of kinetic equations leads to a drift-diffusion type macroscopic system where the diffusion processes dominate the behavior of the solutions.

In the hyperbolic (or high-field) limit the influence of the diffusion terms is of lower (or equal) order of magnitude in comparison with other convective or interaction terms and the models consist of linear or nonlinear hyperbolic equations for the local density.

Finally the use of kinetic models coupled with deterministic thermostats has been recently proposed for the modeling of complex biological systems subjected to external force field, such as a vaccine, but constrained to keep constant the total energy; see [54].

## 5. Multiscale Methods

From the computational point of view, there are methods employed in other field of science that can potentially be employed in biology [11]. These are the Quasi-continuum, the hybrid quantum mechanics-molecular mechanics, the equation-free, the heterogeneous, the multigrid, the multiscale agent-based modeling, the multiscale numerical scheme, and the adaptive tabulation approach. Although we do not describe them here for brevity (suggesting reference [11] as a good starting point), we care to say that despite the fact that each of these has been efficiently applied in a specific problem domain and each has its pro and contra in terms of computational efficiency, none of them has emerged as the multimethod to be used to model biological phenomena.

One example of multiscale approach we care to give more details on is the one we have used to set up a model of (type I) hypersensitive phenomena. According to what just said, it can be classified as a multiscale agent-based model. It consists in an agent-based formulation of the cell-cell/molecules interaction pertaining to hypersensitive responses to a generic allergen in which a detailed gene regulation dynamics is modeled by means of a Boolean network [55] (other approaches, as the use of a system of ordinary differential equations, would work as well [56]). The two levels (the intra- and the inter-cellular) are integrated in a quite intuitive way. For each T lymphocyte, the intracellular gene regulation is driven by the extracellular cytokine concentration (consider it as the* cell input*). On the other hand, the transcription of certain genes can drive the differentiation of the cell and also the production of other cytokines (i.e., the* output*) which influences the overall immune dynamics [8].

What makes this approach appealing is that omics data can effectively be integrated with cellular level data largely available, making a genetic-cause/phenotypic-effect analysis possible [57]. The kind of information clinicians is looking after. Moreover, the two levels of descriptions (the gene regulation through networks) and the intercellular dynamics of the immune response can be developed independently one from another and later put together to account for a more elaborate description of the same, or of other, phenomena. An example would be a detailed description of T helper differentiation in four phenotypes (Th1, Th2, T regulatory, and Th17) [58] which is at the core of, for example, inflammation phenomena, with an agent-based simulation of the immune response not just of infective pathogens or allergens, but also of inflammation and emergence of type 2 diabetes [59, 60].

Other works also incorporated networks or ODEs in agent-based models. See for example, [61] in which the authors discuss the combination of ODEs for chemokine receptor internalisation with agent-based models of lymphocytes in the context of tissue instability in arthritis. Also in [62] the authors describe an approach in which they combine the molecular, the cellular, and the tissue scale in a spatial model of the intestinal crypt. Moreover in [63] Perfahl et al. discuss the domain size effects in the context of vascular tumors in a 3D agent-based approach combined with a reaction-diffusion system.

Kirschner et al. have provided different examples of multiscale immune simulation combining the agent-based paradigm to represent one level of description (i.e., the cellular mesoscopic level) combined to ordinary differential equations. In [64] the authors describe the immune response to M. Tuberculosis representing cells as agents and describing the time-dependent processes essential to antigen processing and presentation by means of ordinary differential equations.

In another work [65], the same authors present an approach for integrating information over relevant biological and temporal scales to generate such a representation for major histocompatibility complex class II-mediated antigen presentation. They then show how this kind of models can be used to suggest new mechanisms and strategies for treatment and vaccines.

When both stochastic fluctuations and spatial inhomogeneity must be included in a model simultaneously, the resulting computational demand quickly becomes overwhelming. In this case it would be useful to use an approach based on coarse-graining methods which turns out to be essential for realistic multiscale models. For instance in [66], the authors present an algorithm for simulation of stochastic, spatially inhomogeneous reaction-diffusion kinetics coupled to coarse-grained fields described by (stochastic or deterministic) partial differential equations (PDEs). They successfully used this method to model cell signaling dynamics in spatially inhomogeneous environments and under the influence of external fields.

### 5.1. General Purpose Integration Methods

When developing a multiscale approach there are few aspects that need to be taken into account. In general, the time scales on which the lower-level processes occur are much faster than those on which the higher-level processes occur. Usually the lower-level processes can be assumed to occur instantaneously and can therefore be included as a representation of some kind of field at the higher level [6]. When we consider joining independent models of processes that occur on different scales, it is enticing to simply couple existing components (i.e., software) for the separate models to one another. This way to proceed does not consider how inaccuracies in the values of the variables that are passed between the two models may affect the combined model. In order to prevent these inaccuracies from occurring one should consider the whole as a single model rather than the combination of two simpler ones. For instance, we can consider that a microscopic simulator at the cellular level can be coupled with the description of the intracellular signaling activating a specific cellular pathway. In this example the differentiation of T lymphocytes into the phenotypes Th1, Th2, Treg, and Th17 is described at a cellular level by means of individual entities (e.g., agent-based) whereas the gene regulation is described by a system of differential equations describing activation level of each gene of the gene network represented with the following equation: *dx*_*i*_/*dt* = (−*e* ^−*Ch*^ + *e* ^−*h*(*ω*_*i*_−*C*)^)/((1 − *e* ^*Ch*^)(1 + *e* ^−*h*(*ω*_*i*_−*C*)^)) − *γ*_*i*_ *x*_*i*_, where *t* is the time, *x*_*i*_ is the activation level of the *i*th gene, *ω*_*i*_ and *γ*_*i*_ are parameters relative to the network topology, and *C* and *h* are constants [55]. Here the lower level description of gene activation is determined at each upper-level time step by solving the system of ODEs and the cell differentiation is executed at the upper level on the basis of the information coming from the gene expression levels. This procedure is iteratively executed at each time step and for each lymphocyte.

From a computational perspective the multiscale nature of innovative models has prompted the important issue of reusability of available published models targeting a single scale. The Physiome project [17, 23] is a prominent effort aiming at solving this problem by developing a framework for the modeling of the “whole” human body. As part of that initiative, the markup language CellML was introduced with the aim of establishing a worldwide adopted standard in the development of cellular levels that are modeled as sets of ODEs [67]. Similarly, FieldML has been defined to model processes on the tissue and organ level that are represented as sets of PDEs [68]. Systems biology markup language (SBML) [69] has been proposed and is now beginning to make a significant impact on the modeling community as a means to exchange models. However, neither CellML nor SBML includes explicit directives to deal with the problem of implementing a multiscale computational model. To solve this important issue, however, there are some attempts, for example, MML [70].

A framework that is devoted to the systems biology community with the target of easy model interoperability is represented by the systems biology workbench [71], a high performance, open-source software infrastructure that allows heterogeneous application components written in diverse programming languages and running on different platforms to communicate and use each others' capabilities via a message system.

## 6. Concluding Remarks

In the study of complex biological phenomena it is necessary to develop a unified view of the various mechanisms in action and of the causal relationships among different parts of that complex system, [4, 7]. In this paper we have briefly described the problems faced when one wants to link mathematical or computational models across different time and length scales.

In many areas of biology and physiology, multiscale and multiphysics models are very much acclaimed, although there exists an abundant literature for multiscale models in science and engineering domains [72], a lot remains to be done in terms of translating these mathematical theories and methodologies to the domains of biology and physiology [73–75].

A key unsolved issue is how to represent appropriately the dynamical behaviors of a high-dimensional model of a lower scale by a low-dimensional model of a higher scale, so that it can be used to investigate complex dynamical behaviors at even higher scales of integration [14]. Indeed, the ultimate goal of multiscale modeling is not just about developing models at different scales but to link them in a consistent manner so that the information from a lower scale can be carried into the simplified model of a higher scale.

The use of different modeling approaches introduces gaps among scales. Multiscale modeling, besides modeling the system, needs to address the issue of how to bridge the gaps between different methodologies and between models at different scales. Unfortunately, there is no specific or simple way to tell how to achieve this objective, but there are empirical principles and methods that can be of help.

In the study of the immune system and related pathologies, one method for constructing multiscale models that has been used by various authors resorts to agents to represent the mesoscopic level of cells of the immune system (i.e., the multicellular rule-based modeling in [76]) while employing ordinary differential equations to describe the intracellular events as intracellular signalling and partial differential equations to describe cytokines diffusion at the extracellular or tissue scale. Level coupling is then performed in a quite straightforward way using concentrations as input variables to the cellular agents. Whereas modeling intracellular events can be implemented in many ways (e.g., Boolean networks or other generic decision mechanisms) without explicitly including the variable “space” for computational reasons (but mainly for simplicity), the diffusion of cytokines (or, another example, cells relocation between anatomical compartments), is a spatial phenomenon in character. This can be modeled as a continuous (by means of PDEs) or as a discrete process (e.g., lattice gas) for which the computational efficiency is the major limiting factor.

The goal of computational systems biology is to consider a biological system from a holistic perspective and use both experiments and modeling to reveal how the system behaves [4, 77]. Multiscale models able to exploit laboratory and clinical data at different levels can potentially bridge knowledge gaps between what is observed at the gene/molecular level and the clinical evolution of complex diseases [11].

Finally, by integrating these models with detailed monitoring data from emerging body-sensor technology [78], health care practitioners could be supported in taking diagnosis and suggesting optimal therapeutic regimens thus promoting the much acclaimed patient-specific view of modern health care systems.
